# Regulation of H3K4me3 breadth and MYC expression by the SETD1B catalytic domain in MLL-rearranged leukemia

**DOI:** 10.1038/s41375-025-02638-y

**Published:** 2025-05-08

**Authors:** Shintaro Izumi, Ko Ohtani, Makoto Matsumoto, Seito Shibata, Bahityar Rahmutulla, Masaki Fukuyo, Mitsutaka Nishimoto, Hideo Miyagawa, Emiko Sakaida, Koutaro Yokote, Issay Kitabayashi, Kimi Araki, Atsushi Kaneda, Takayuki Hoshii

**Affiliations:** 1 Department of Molecular Oncology, Graduate School of Medicine, Chiba University, Chiba-shi, Chiba, Japan; 2 Department of Endocrinology, Hematology and Gerontology, Graduate School of Medicine, Chiba University, Chiba, Japan; 3 Department of Hematology, Osaka Metropolitan University Graduate School of Medicine, Osaka, Japan; 4 Preventive Medicine and Environmental Health, Graduate School of Medicine, Osaka Metropolitan University, Osaka, Japan; 5 Oncology Innovation Center/ Center for Translational Research, Fujita Health University, Aichi, Japan; 6 Division of Developmental Genetics, Institute of Resource Development and Analysis, Kumamoto University, Kumamoto, Japan; 7 Center for Metabolic Regulation of Healthy Aging, Kumamoto University, Kumamoto, Japan; 8 Health and Disease Omics Center, Chiba University, Chiba, Japan

**Keywords:** Acute myeloid leukaemia, Cancer epigenetics, Genomics

## Abstract

Histone H3 lysine 4 trimethylation (H3K4me3) is abundant in mixed-lineage leukemia-rearranged (MLL-r) acute myeloid leukemia (AML) cells; however, the responsible enzymes and their roles remain unclear. This study aimed to identify the modifier responsible for high H3K4me3 modification in MLL-r leukemia and its downstream targets essential for the cell proliferation. Here, we performed a CRISPR-tiling screen against known H3K4 methylation modifiers in an MLL-r AML model. Disrupting the SETD1B catalytic SET domain caused depletion of FLT3-ITD or Nras^G12D^-expressing AML cells, and gene expression downregulation, particularly in the MYC pathway. SETD1B SET domain loss results in a significant decrease in H3K4me3 breadth. Exogenous MYC expression or disrupting H3K4 demethylase KDM5C significantly restored growth defects in SETD1B SET domain-mutant cells. These data indicated that SETD1B was required for H3K4me3 breadth and MYC expression. Thus, a thorough understanding of SETD1B-mediated H3K4me3 breadth is critical for developing markers and therapies for MYC-dependent leukemia subtypes.

## Introduction

Acute myeloid leukemia (AML) with Fms-like tyrosine kinase-3 internal tandem duplication (FLT3-ITD) is strongly correlated with recurrence and poor prognosis [1, 2]. FLT3-ITD cause constitutive RAS signaling pathway activation and alter the epigenetic landscape and gene expression in leukemia [3–5]. FLT3 mutations (D835 and ITD) are detected in patients with leukemia with mixed-lineage leukemia-rearrangement (MLL-r), caused by a translocation of H3K4 methyltransferase (H3K4HMT) MLL1 (also known as KMT2A) [6, 7]. FLT3-ITD cooperates with MLL-AF9 (also called KMT2A-MLLT3) to accelerate AML onset in a mouse model [8]. Thus, these mutations may cooperatively induce an aberrant epigenetic landscape during leukemogenesis. However, little is known about the interaction between FLT3-ITD and H3K4HMTs in AML cells.

MLL1 fusion oncoprotein lacks the catalytic SET domain, and the MLL1 SET domain from wild-type alleles is dispensable in MLL-r leukemia cells [9]. Previous research has reported the redundant roles of MLL1/2 (KMT2A/B) and the indispensable role of MLL4 (KMT2D) in MLL-r AML cells [10–12]. Histone H3 lysine 4 tri-methylation (H3K4me3) is a histone modification associated with active transcription, and this is enriched around the transcription start site (TSS) of active genes and the gene body of stably expressed genes [13–15]. In MLL-r AML, higher H3K4me3 levels are observed in leukemia-initiating cells compared to differentiated cells; however, little is known about the role of H3K4HMT responsible for H3K4me3 [16]. Multiple histone methyltransferases and demethylases modify H3K4 methylations in mammals [17–21]. Set1 catalyzes all H3K4 methylations in yeast, and the yeast orthologs with the most similar protein domain structures are SETD1A/B in mammals [22]. We previously determined that the catalytic function of SETD1A is dispensable for H3K4me3 and cell proliferation in MLL-r AML cells [23, 24]. Thus, other H3K4HMTs may redundantly or predominantly regulate H3K4me3 in AML cells. In this study, we aimed to identify the H3K4HMT modifier responsible for high H3K4me3 modification in MLL-r leukemia and its downstream targets essential for the cell proliferation.

## Materials and methods

### Generation of *Setd1a SET*^*flox*^ mice and AML cell lines

*Setd1a SET*^*flox*^ mice were generated by introducing the Cas9 protein (317–08441; Nippon Gene, Tokyo, Japan), tracrRNA (GE-002; FASMAC) and synthetic crRNA (FASMAC), and single-stranded oligodeoxynucleotides (ssODN) into C57BL/6N fertilized eggs using electroporation. The synthetic crRNAs were designed to target the 16th and 17th introns of *Setd1a*, and two ssODNs were used as homologous recombination templates to insert *lox*P sequences (Supplementary Table 1). The electroporation solution contained 10 μM of tracrRNA, 10 μM of synthetic crRNA, 0.1 μg/μL of Cas9 protein, and 1 μg/μL of ssODNs in Opti-MEM I Reduced Serum Medium (Thermo Fisher Scientific, Waltham, MA, USA). Electroporation was performed as previously described [25, 26]. After electroporation, the embryos were cultured overnight and then transferred into the oviducts of foster mothers at the two-cell stage. Wild-type, SET^flox^ (SF), and SET^del^ (SD) alleles were genotyped using primers (Supplementary Table 1). The AML cell line was established from bone marrow LSK (lineage-, Sca-1+, and c-KIT+) cells by transducing MLL-AF9-neo, FLT3-ITD-DsRed, CreER^T2^-Puro, and Cas9-Blast constructs using retrovirus/lentivirus vectors, as previously described [23]. All plasmids used in this study are listed in Supplementary Table 1.

### Cell lines and cell culture

293T, MOLM-13, U937 and K562 cell lines were obtained from the ATCC (Manassas, VI, USA). Plat-E cells were obtained from Dr. Toshio Kitamura. RN2 cells were obtained from Dr. Scott W. Lowe. Murine MLL-AF9 leukemia (MA9) cells and Cas9-expressing cell lines were established previously [23]. All media were supplemented with 10% fetal bovine serum (FBS) and 1% penicillin-streptomycin. 293T and Plat-E cell lines were maintained in Dulbecco’s Modified Eagle Medium. MA9 and ∆SET cells were maintained in RPMI1640 medium containing 10 ng/mL recombinant murine interleukin-3 (IL-3). MOLM-13, U937, RN2, and MA9/FLT3-ITD cells were cultured in RPMI1640 medium. K562 cells were cultured in Improved Minimum Essential Medium. Cells were washed twice and re-suspended in RPMI1640 medium for cytokine depletion.

### cDNA expression in leukemia cells

*SETD1B* cDNA was cloned into the pLEX_305-N-dTAG vector. The vector was transiently transfected into 293T cells with psPAX2 and pMD2.G packaging plasmids. The lentivirus in the supernatant was infected in MOLM-13 cells and selected using 1 µg/mL puromycin for 7 days. *Myc* cDNA was cloned into the pMSCV-PGK-hygro vector, and retroviruses were generated in Plat-E cells. *Setd1b* wild-type or mutated MA9/FLT3-ITD cells were infected with the retrovirus and selected using 1 mg/mL hygromycin for 14 days.

### CRISPR

sgRNA was designed using CRISPick (http://broad.io/crispick), and a pooled 5730 sgRNA oligo library was synthesized by TwistBioscience (San Francisco, CA, USA) (Supplementary Table 1). The library was amplified using polymerase chain reaction (PCR) and inserted into the pLKO5.sgRNA.EFS.GFP vector. After titration of viral particles, 1 × 10^7^ murine *Setd1a*^*SF/SF*^/MLL-AF9-neo/FLT3-ITD-DsRed/CreER^T2^-Puro/Cas9-Blast leukemia cells were infected at a multiplicity of infection of 0.3. Then, 3 × 10^6^ GFP-positive cells were sorted using the cell sorter SH800 (Sony, Minato City, Tokyo, Japan) on day three post-infection. Sorted cells were treated with tamoxifen to delete the SETD1A SET domain. Three million cells were passaged every three days, and genomic DNA was harvested on day seven post-treatment. The sgRNA sequences in the genomic DNA were amplified with the primers listed in Supplementary Table 1. The PCR products were purified using AMPure XP beads and quantified using QuantiFluor dsDNA system (Bio-Rad, Hercules, CA, USA). Libraries were pooled and sequenced using Illumina NovaSeq 6000 (Illumina, San Diego, CA, USA). Data were analyzed using PoolQ-3.3.2. Graphs were visualized using Prism 9 software (GraphPad software). Human doxycycline-inducible Cas9-expressing cell lines were established in our previous study [23]. Doxycycline at a concentration of 1 µg/mL was used in doxycycline-inducible Cas9 cells. For individual gene knockout, sgRNAs were subcloned into pLKO5.sgRNA.EFS.GFP, pLKO5.sgRNA.EFS.tRFP657 or plenti-sgRNA hygro vectors. sgRNA/GFP-expressing cells were sorted using a cell sorter SH800 on day three post-infection. Cells expressing sgRNA/hygro were selected using 1 mg/mL hygromycin for 7 days. For the in vivo AML model, 1 × 10^4^ purified murine AML cells were transplanted into lethally irradiated (9.5 Gy) syngeneic 10-week-old female C57BL/6J recipient mice and 5 × 10^5^ normal bone marrow mononuclear cells, following an animal protocol approved by Chiba University (No. 5–54).

### Generation of *Setd1b* ∆SET cells

MLL-AF9/Cas9 leukemia cells were infected with the FLT3-ITD-DsRed retroviral vector and cultured without IL-3 to select cytokine-independent cells. The established cells were cultured in RPMI medium containing 10 ng/mL IL-3 for one week and infected with *Setd1b* SET domain-targeting sgRNA (sgSetd1b SET-1) lentivirus vectors. Single-cell cloning was performed using the limiting dilution method. The selected clones were expanded, and the deletion allele was confirmed using PCR with specific primers, followed by DNA Sanger sequencing by ABI 3130 genetic analyzer (Thermo Fisher Scientific).

### Apoptosis, cell cycle, differentiation, and growth

Apoptosis was evaluated using Annexin V Apoptosis Detection Kit (eBioscience, San Diego, CA, USA). For cell cycle analysis, cells were fixed in 50 µL of 4% paraformaldehyde at 37 °C for 10 min and then permeabilized with 450 µL cold MetOH for 30 min on ice. The cells were washed twice with 5% FBS/phosphate-buffered saline (PBS) and stained with Alexa Fluor 647-conjugated anti-Ki-67 antibody (BD Biosciences, Franklin Lakes, NJ, USA; 558615) for 1 h at room temperature. The cells were washed again, counterstained with DAPI in 5% FBS/PBS, and analyzed using BD FACS Canto II (BD Bioscience). For cell differentiation, the cells were attached to glass slides using by Cytospin (Thermo Fisher Scientific), and stained with Jorvet Dip Quick stain kit (Thermo Fisher Scientific). The GFP or tRFP657 positive cell ratio or absolute cell numbers were measured using CytoFLEX (Beckman Coulter, Brea, CA, USA) for cell growth assays. Cells were cultured with or without IL-3 or recombinant murine granulocyte-macrophage colony-stimulating factor (GM-CSF) to analyze the effects of cytokines.

### Colony assay

Cells were cultured in methylcellulose media (MethoCult M3231; STEMCELL Technologies, Vancouver, Canada) containing 10 ng/µL IL-3 (Peprotech, Rocky Hill, NJ, USA) for 7–10 days. Colonies were counted under a microscope. For the replating assay, cells in methylcellulose medium were washed twice with 5% FBS/PBS, and the counted cells were cultured again in methylcellulose medium containing IL-3.

### Western blotting

Lysates were prepared, separated, and transferred to a polyvinylidene difluoride membrane as previously described [24]. Histones were extracted using the Histone Extraction Kit (Abcam). Blots were blocked with 5% milk in TBS-T for 30 min at room temperature and incubated with primary antibodies (Supplementary Table 1) at 4 °C overnight. Immunocomplexes were labeled with horseradish peroxidase-conjugated secondary antibodies and visualized using Amersham ECL Prime (Cytiva, Marlborough, MA, USA). The signals were detected using ChemiDoc Touch MP (Bio-Rad) and quantified using ImageJ/Fiji software.

### RNA analyses

Total RNA was purified using RNeasy Mini Kit with DNase set (Qiagen). cDNA was synthesized using ReverTra Ace qPCR RT Master Mix (TOYOBO, Osaka, Japan) and quantified using TaqMan Probes or specific primers (Supplementary Table 1) with CFX96 Touch real-time PCR detection system (Bio-Rad). For RNA-seq, ERCC ExFold RNA Spike-In Mixes (Thermo Fisher Scientific) were added for normalization before library preparation. RNA-seq libraries were prepared using TruSeq Stranded mRNA Sample Prep Kit (Illumina). The DNA library was validated using TapeStation (Agilent Technologies, Santa Clara, CA, USA) and quantified using QuantiFluor dsDNA system (Promega, Madison, WI, USA). Libraries were pooled and sequenced using Illumina NextSeq 500 and NovaSeq 6000. Data were analyzed using HISAT2 and Cufflinks software. The list of genes of interest was analyzed using Enrichr tool (https://amp.pharm.mssm.edu/Enrichr/). The data were visualized in Python using Matplotlib and Seaborn.

### ChIP and CUT&Tag analyses

The cells were fixed, sonicated, and immunoprecipitated for ChIP analyses as previously described [24, 27]. ChIP DNA was quantified using quantitative PCR with specific primers (Supplementary Table 1). Libraries were prepared and sequenced as described previously. CUT&Tag libraries with anti-MYC antibodies (Cell signaling #13987) were prepared as described previously [27]. Data were mapped to the reference genomes (mm10 or hg19) using Bowtie2, and duplicated reads were removed using Picard tools. Peak calling was performed using HOMER software. Data were visualized using Integrative Genomics Viewer, deepTools within Galaxy, Java TreeView, and Python with Matplotlib and Seaborn. Alterations in H3K4me3 breadth were calculated using the subtraction tool on Galaxy. The polymerase (Pol) II traveling ratio was calculated as described previously [28, 29]. Gene annotation of ChIP-seq peaks was performed using the Genomic Regions Enrichment of Annotations Tool using the basal plus extension method.

### Ethical approval and consent to participate

All adult AML patient samples were collected by Osaka Metropolitan University and Chiba University in accordance with the Declaration of Helsinki. Informed consent was obtained from all patients, and the study was approved by the research ethics boards of the Graduate School of Medicine, Osaka Metropolitan University (Approval No. 2023-147), Chiba University Hospital (Approval No. HS202209-08), and the Graduate School of Medicine, Chiba University (Approval No. 1226).

### Quantification and statistical analyses

Statistical analyses were performed using Prism 9 and EZR software. We performed the Student’s *t*-test or one-way ANOVA, followed by Tukey’s test for statistical comparisons. Survival curves were compared using the Log-rank (Mantel-Cox) test. Correlations were analyzed using the Spearman’s test. *P* values lower than 1e-15 in the EZR are defined as <1e–15. Error bars in all data represent the standard deviation. For randomization to pick up H3K4me3 low/negative genes, select random lines tool in Galaxy was used.

## Results

### SETD1B is an essential H3K4 methyltransferase in FLT3-ITD or Nras^G12D^-expressing MLL-r leukemia cells in vitro

**Fig. 1:**
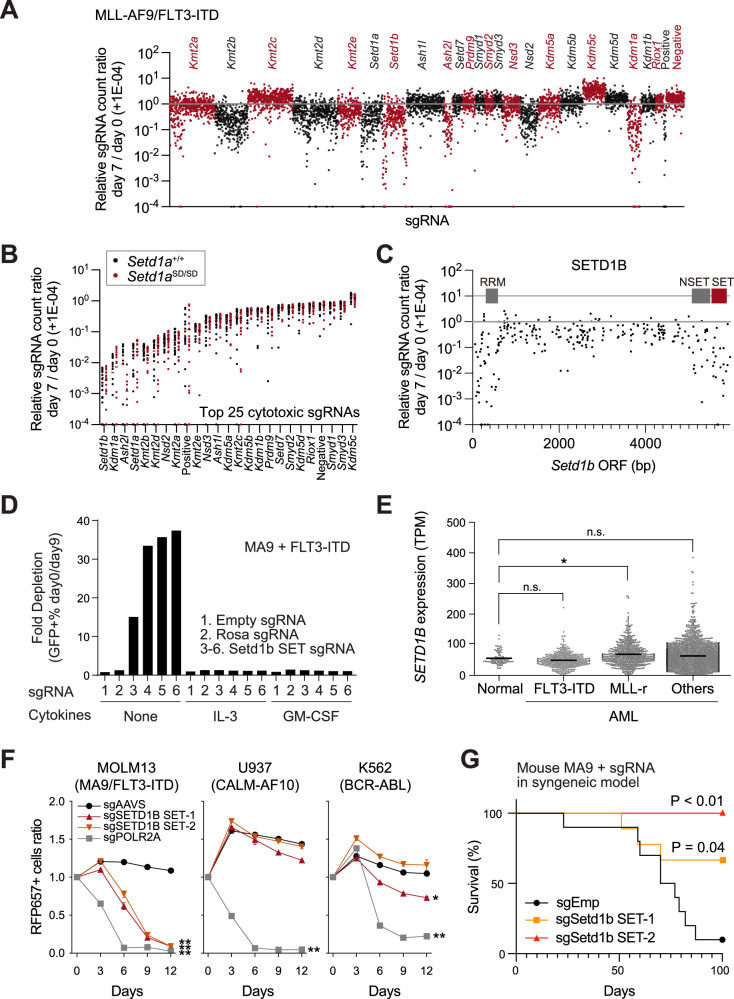
The catalytic domain of SETD1B, but not SETD1A, is required for acute myeloid leukemia (AML) cell proliferation. **A** The results from the pooled CRISPR-tiling screen against H3K4HMT genes in MLL-AF9/FLT3-ITD/Cas9 AML cells. **B** The top 25 cytotoxic sgRNAs were selected from each gene. *Setd1b* is shown on the far left as the most effective target. **C** The relationship between known domains and sgRNAs against *Setd1b*. Dots represent the relative sgRNA count ratio from the CRISPR tiling screen in **A**. **D** Negative selection experiments evaluating Setd1b SET sgRNA in MLL-AF9/FLT3-ITD cells were performed with or without cytokines. **E** *SETD1B* expression levels in human AML patient dataset from TARGET-AML. Data from normal bone marrow (*n* = 88), FLT3-ITD AML (*n* = 392), MLL-r AML (*n* = 554) and other AML types (*n* = 1811) were used. **F** SETD1B SET domain-targeting sgRNA was transduced into MOLM-13, U937, and K562 cells, and competitive growth assay was performed. POLR2A sgRNA was used as the positive control. **G** Survival of the transplanted recipient mice with Setd1b SET domain targeting sgRNA-expressing MLL-AF9 leukemia cells (*n* = 9–10 per group). *P < 0.05, **P < 0.01. n.s. no significance.

We performed a CRISPR-tiling screen targeting known H3K4 methylation modifiers in mouse MLL-AF9/FLT3-ITD/Cas9 AML cells to identify the H3K4 methylation modifiers implicated in MLL-r/FLT3-ITD AML (Fig. 1A). We observed a decrease in cell number in AML cells transfected with sgRNA-directed *Setd1b*, *Kdm1a*, and *Ash2l* (Fig. 1A–C, Supplementary Fig. 1A, B). *Setd1a* also ranks as the fourth most important factor in this study, however the catalytic SET domain is dispensable in MLL-r AML (Fig. 1A) [23]. To confirm the role of the SETD1A SET domain in MLL-r/FLT3-ITD AML, we generated mice with floxed *Setd1a* exon 17, allowing deletion of the SETD1A S-adenosyl methionine-binding region (Supplementary Fig. 1C), and established a leukemia cell line by transducing retrovirus-based expression vectors for MLL-AF9, FLT3-ITD, and CreER^T2^ into bone marrow-derived LSK cells (Supplementary Fig. 1C, D). Deleting exon 17 did not significantly affect the colony formation ability in vitro (Supplementary Fig. 1E), indicating that the endogenous SETD1A catalytic function is dispensable in AML cell proliferation. CRISPR-tiling screening revealed that H3K4 methylation modifiers have a non-redundant function with the SETD1A SET domain in AML (Fig. 1B, Supplementary Fig. 1F). Furthermore, these results indicated that the SETD1B SET domain plays an indispensable role in FLT3-ITD-positive AML cell proliferation, irrespective of the presence or absence of the SETD1A SET domain (Fig. 1B, C).

In our previous investigation, we reported a dispensable role of SETD1B in murine MLL-AF9 leukemia cells [23]. Therefore, we performed the CRISPR assay against the SET domain of MLL family genes in two other murine MLL-r leukemia models to confirm the effect of SETD1B SET domain disruption. One was the MA9 cell line, which has only the MLL-AF9 transgene, and the other was the RN2 cell line, which has MLL-AF9 and Nras^G12D^ transgenes [30]. MA9 cells showed resistance against Setd1b sgRNA (Supplementary Fig. 1G). In contrast, RN2 cells showed a strong sensitivity to Setd1b sgRNAs (Supplementary Fig. 1H). MA9 cells, unlike FLT3-ITD-positive or RN2 cells, require the addition of IL-3 or GM-CSF. These results suggest that activating the cell signaling pathway modulates the effects of SETD1B disruption.

We transduced FLT3-ITD into MA9 cells and monitored their sensitivity to Setd1b sgRNA with or without cytokines to examine the effects of oncogenic signaling and cytokines on MLL-r leukemia cells. First, FLT3-ITD transduced MA9 cells were grown without IL-3 and were sensitive to Setd1b sgRNA (Fig. 1D). In contrast, re-administering IL-3 or GM-CSF reduced Setd1b sgRNA sensitivity (Fig. 1D). Although GM-CSF showed a stronger effect on Setd1b sgRNA resistance than IL-3 did, IL-3 and GM-CSF induced Setd1b sgRNA resistance in RN2 cells (Supplementary Fig. 1I). The *SETD1B* expression was significantly elevated in patients with MLL-r leukemia (Fig. 1E). SETD1B dependency was also observed in the MOLM-13 human MLL-r/FLT3-ITD leukemia cell line but not in the U937 and K562 non-MLL-r leukemia cell lines (Fig. 1F). In the syngeneic murine AML model, Setd1b sgRNA expression decreased MLL-r leukemia cell propagation in vivo, even without FLT3-ITD or Nras^G12D^ expression; thus, SETD1B requirement increased in the in vivo environment (Fig. 1G). These findings imply that SETD1B catalytic function is required in vitro and in vivo, particularly in cytokine-deprived environments.

**Fig. 2:**
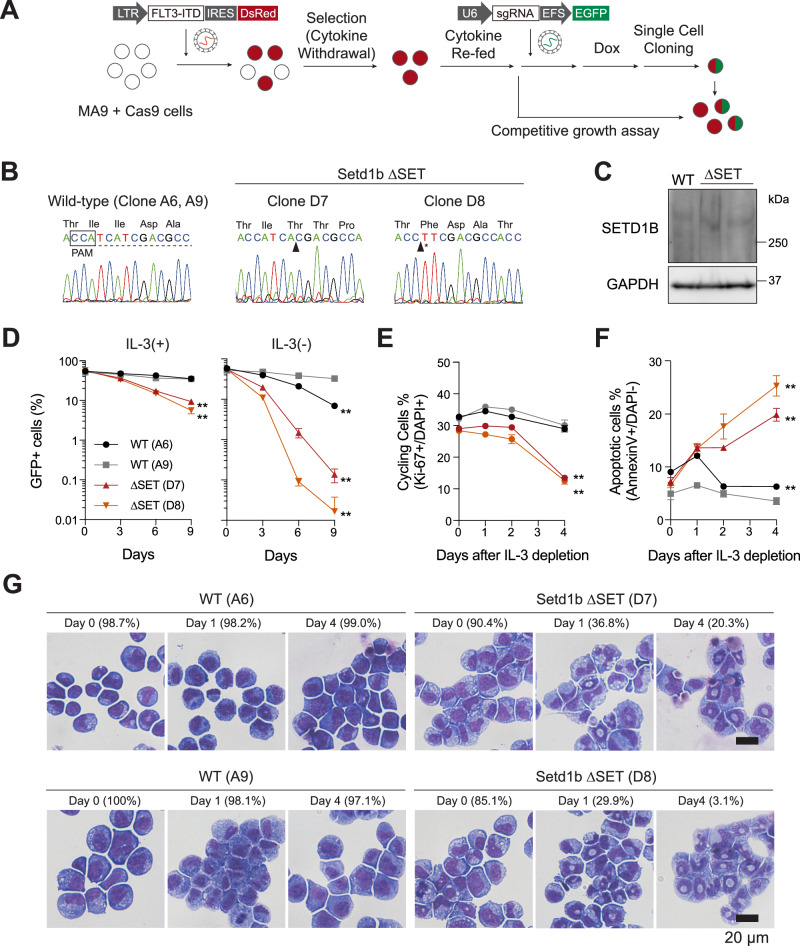
SETD1B promotes the cytokine-independent growth of MLL-r AML cells. **A** Scheme showing the establishment of SETD1B SET domain mutant MLL-AF9/FLT3-ITD AML cell clones. **B** The DNA sequence of *Setd1b* in AML cell clones. **C** Western blot analysis of endogenous SETD1B protein expression. **D** Proliferation of *Setd1b* mutated cells in competitive growth assay with (left) or without (right) interleukin-3 (IL-3). **E** The proportion of cell cycling (Ki-67 + /DAPI + ) cells, (**F**) apoptotic (Annexin V + /DAPI-) cells, and **G** morphological changes in IL-3 depleted AML cells are shown. The percentages of blast cells are shown. Representative data from two independent experiments with three biological replicates are shown. Scale bar: 20 µm. **P < 0.01.

To evaluate the role of the SETD1B catalytic domain, we established single-cell-derived SETD1B SET domain mutant clones (D7, D8) using CRISPR-Cas9 after re-administering IL-3 (Fig. 2A). D7 had a frameshift mutation, while D8 exhibited an in-frame deletion of two isoleucine residues in the SET domain. Despite the mutations, ∆SET clones expressed the comparable levels of SETD1B protein (Fig. 2B, C). These clones could expand in media containing IL-3 with a lower cell proliferation rate than parental cells. However, they showed a striking decrease in cell number, and cell cycling, an increase in apoptosis and granulocyte differentiation after withdrawing IL-3 treatment (Fig. 2D–G). Our results indicate that SETD1B is an essential H3K4 methyltransferase for cytokine-independent cell proliferation in MLL-r leukemia cells.

### SETD1B and cytokines cooperatively regulate MYC expression

**Fig. 3:**
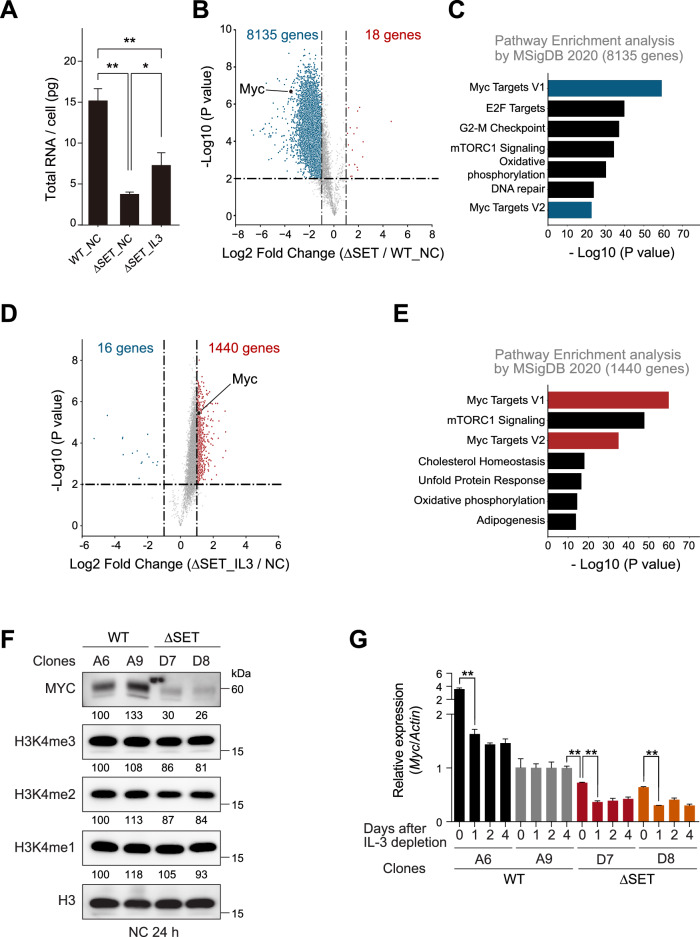
SETD1B and IL-3 signaling cooperate for MLL-r AML cell growth through MYC expression. **A** The total RNA amount in WT_NC (wild-type, no cytokine), ∆SET_NC (∆SET, no cytokine) and ∆SET_IL3 (∆SET, IL-3) AML cells. **B** RNA-seq analysis with a spike-in control was performed for WT_NC and ∆SET_NC cells. **C** Pathway analysis of downregulated genes in ∆SET_NC cells using Enrichr. **D** RNA-seq analysis with a spike-in control was performed for ∆SET_IL3 and ∆SET_NC cells. **E** Pathway analysis of upregulated genes in ∆SET_IL3 cells using Enrichr. **F** Western blotting analysis of MYC and histone modifications (H3K4me1/2/3) in *Setd1b* mutant cells. Cells were cultured for 24 h without IL-3 (no cytokine, NC) before use. The numbers below represent a semi-quantitative analysis of the indicated proteins. **G** The relative expression of *Myc* in WT and ∆SET cells at the indicated days after the IL-3 depletion. *P < 0.05, **P < 0.01.

We then investigated the changes in gene expression profiles using RNA-seq. Because of the reduced total RNA content in the SETD1B mutants, we used RNA spike-in controls in this study (Fig. 3A). Consistent with the RNA content, ∆SET cells showed a global reduction in RNA, and pathway enrichment analysis revealed that SETD1B loss decreases *Myc* expression (Fig. 3B, C). In contrast, administering IL-3 restored global RNA production and *Myc* expression and upregulated gene expression associated with the MYC signature (Fig. 3A, D, E). MYC is essential for RN2 cell proliferation and cytokine-independent myeloid progenitor cell growth [30, 31]. ∆SET cells showed decreased MYC expression and slightly decreased global H3K4me2/3 modification levels (Fig. 3F). After depleting IL-3, high *Myc* expression was maintained in control cells; however, *Myc* expression was significantly decreased in ∆SET cells (Fig. 3G). Notably, *Myc* downregulation was observed in the A6 clone after IL-3 depletion and in ∆SET cells even before IL-3 depletion (Fig. 3G). This observation aligns with sustained IL-3 dependency of the A6 clone and the partial defects in ∆SET cells under IL-3 conditions (Fig. 2D–G). These results indicate that the catalytic domain of SETD1B and cytokines are both required for *Myc* expression and MYC-dependent leukemia cell growth.

### SETD1B SET mutant AML cells show defects in broad H3K4me3 in the gene body

**Fig. 4:**
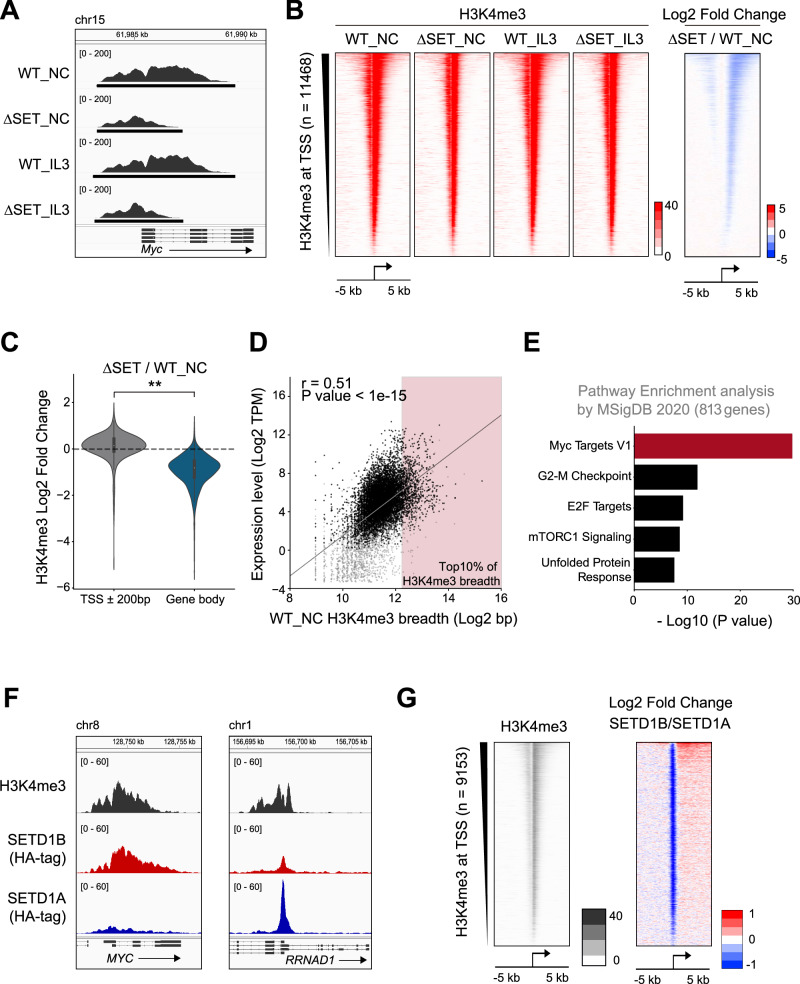
SETD1B promotes the H3K4me3 breadth and MYC expression. **A** H3K4me3 distribution at the *Myc* locus in WT and ∆SET cells in the presence of IL-3 or without cytokines (NC). **B** The heatmaps of H3K4me3 ChIP-seq in WT and ∆SET cells and the log2 fold change of H3K4me3 signals between WT and ∆SET samples without cytokines are shown. **C** The violin plot for the log2 fold change of H3K4me3 signals at the TSS ± 200 bp or gene body (from TSS + 400 to TES) (*n* = 11,468). **D** The correlation between H3K4me3 breadth and the mRNA expression (with transcript per million mapped reads (TPM) > 0.1, grey dots) in AML cells. A total of 8135 downregulated genes in *Setd1b* ∆SET cells are shown in black, and genes with the top 10% of H3K4me3 breadth (*n* = 813) are shown in the red region. **E** Pathway analysis of genes annotated from the top 10% of H3K4me3 breadth (*n* = 813) with decreased H3K4me3 breadth in **D**. **F** Representative browser views of ChIP-seq for H3K4me3, SETD1B and SETD1A at *MYC* and *RRNAD1* loci in MOLM-13 human MLL-r/FLT3-ITD leukemia cells. The *RRNAD1* locus is shown as a representative SETD1A-target gene. **G** The heatmaps of H3K4me3 ChIP-seq and the log2 fold change between SETD1B and SETD1A are shown. **P < 0.01.

SETD1B catalyzes H3K4me3 in vitro [32]; therefore, we performed ChIP-seq analysis for H3K4me3 using SETD1B-mutated AML cells to measure the effects of SETD1B disruption on H3K4me3 distribution. H3K4me3 modification was observed at the *Myc* locus, especially at the gene body of *Myc* in wild-type cells, and the broad H3K4me3 peaks were dramatically reduced in ∆SET cells (Fig. 4A). The reduced H3K4me3 at the gene body of *Myc* was confirmed in the other ∆SET clones and early after SETD1B SET sgRNA transduction (Supplementary Fig. 2A, B). A reduction in H3K4me3 in the gene body was also observed at other loci with broad H3K4me3 peaks (Fig. 4B). IL-3 did not change the width of H3K4me3 positive regions in wild-type cells, but it moderately increased around the TSS in ∆SET cells (Supplementary Fig. 2C, D). Notably, H3K4me3 peaks were retained at the TSS in ∆SET cells; thus, SETD1B promotes H3K4me3 modification at the gene body, but other H3K4HMTs contribute to maintaining H3K4me3 at TSS (Fig. 4B, C). H3K4me3 breadth was positively correlated with the mRNA expression levels in our data (Fig. 4D). The width of reduced H3K4me3 breadth in ∆SET cells was significantly correlated with the width of the original H3K4me3 breadth (Supplementary Fig. 2E). The top 10% of genes with H3K4me3 breadth were enriched in the MYC target signature (Fig. 4E). We also performed ChIP-seq for SETD1B and SETD1A using HA-tagged SETD1B or SETD1A expressing MOLM-13 human MLL-r/FLT3-ITD leukemia cells. SETD1B was broadly distributed on the MYC and other loci with broad H3K4me3 at the gene body; however, SETD1A was generally localized at the TSS (Fig. 4F, G, Supplementary Fig. 2F). SETD1B was enriched in the gene bodies with H3K4me3 breadth (Fig. 4G). These results indicate that SETD1B promotes MYC expression and its downstream targets through catalytic domain function by maintaining H3K4me3 breadth in AML cells.

### The SETD1B SET mutant AML cells can be rescued by MYC expression

**Fig. 5:**
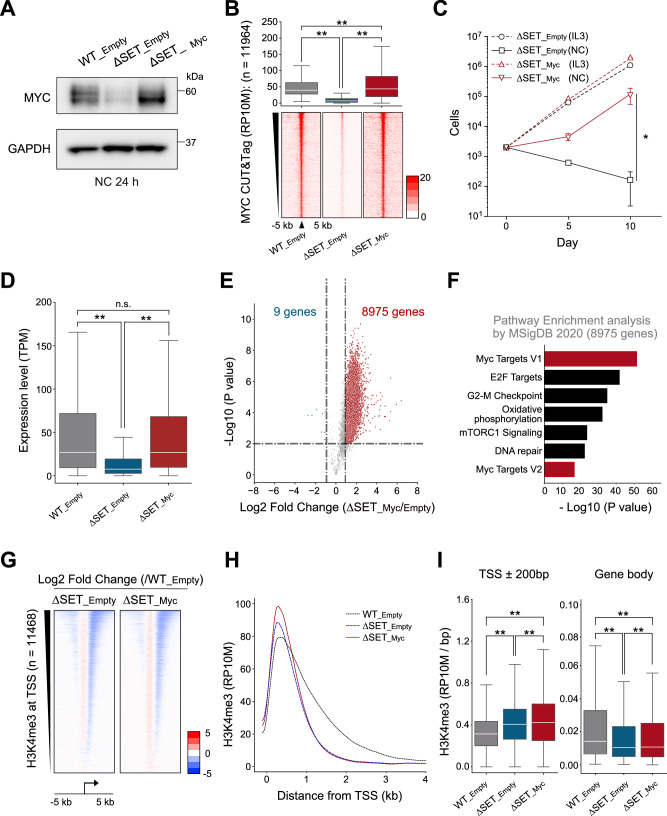
MYC expression rescues the SETD1B ∆SET mutant cell growth. **A** Measurement of endogenous and exogenous MYC protein expression using western blotting. **B** CUT&Tag analysis of the MYC protein. The box plots and the heat maps of MYC peaks are shown. Black arrows indicate the peak center. **C** Cell proliferation assay for MYC-expressing ∆SET leukemia cells with or without IL-3. RNA-seq analysis using a spike-in control was performed. **D** The box plot indicates the restoration of gene expression after MYC expression. **E** The volcano plot for ∆SET_Empty and ∆SET_Myc cells indicates the increased gene expressions. **F** Pathway analysis of the 8975 upregulated genes in ∆SET_Myc cells using Enrichr. **G** The heatmaps of log2 fold change in H3K4me3 signals between WT_Empty and ∆SET_Empty or ∆SET_Myc. **H** The histograms of H3K4me3 ChIP-seq average signals at downstream from TSS (*n* = 813). **I** The boxplots for H3K4me3 levels at the TSS or gene body in WT_Empty, ∆SET_Empty, and ∆SET_Myc cells (*n* = 813). **P < 0.01, *P < 0.05. TSS, transcription start site.

We transduced *Myc* cDNA into ∆SET cells to investigate the role of MYC in SETD1B ∆SET AML cells. As expected, reduced MYC expression and chromatin distribution in ∆SET leukemia cells were restored by exogenous MYC expression (Fig. 5A, B). Notably, MYC expression significantly recovered ∆SET cell proliferation in the IL-3-depleted condition (Fig. 5C). The MYC-rescued ∆SET cells showed higher gene expression levels than empty vector-transduced ∆SET cells, as observed in the IL-3-treated ∆SET cells (Fig. 5D, E). The upregulated genes in the MYC-rescued cells showed a signature similar to that of the pathways enriched in the top 10% of H3K4me3 breadth-associated genes (Fig. 4E), demonstrating that MYC is a crucial downstream target of SETD1B in AML cells (Fig. 5F). In contrast to restoring gene expression in ∆SET cells, reduced broad H3K4me3 signals in the gene body were not reversed by MYC expression (Fig. 5G–I). These data indicated that SETD1B-dependent H3K4me3 is an upstream regulator of MYC in leukemia.

### H3K4me3 regulates RNA Pol II elongation in MLL-r leukemia cells

**Fig. 6:**
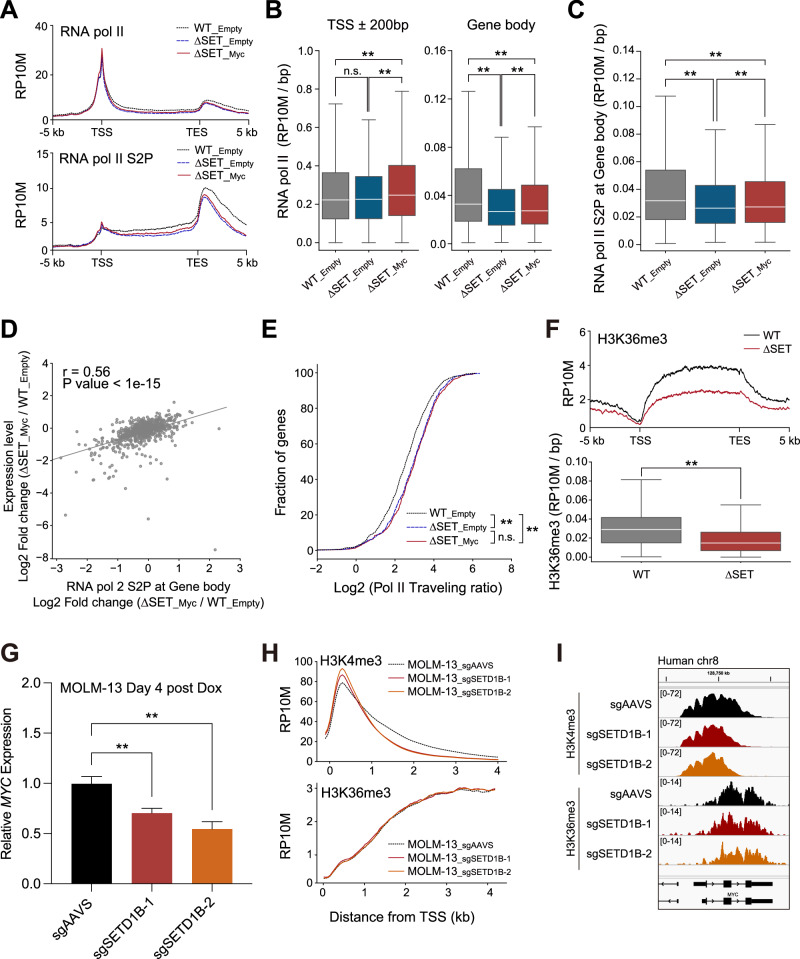
H3K4me3 is required in RNA Pol II elongation in *Setd1b* ∆SET leukemia cells. **A** Histograms of RNA Pol II and CTD Ser2 phosphorylation (S2P) in WT_Empty, ∆SET_Empty, and ∆SET_Myc cells. Gene loci annotated from the top 10% of H3K4me3 breadth were used. **B** Box plots of the RNA Pol II signal. **C** Boxplots of S2P. **D** Correlation between the log2 fold change of S2P in the gene body and mRNA expression in WT_Empty and ∆SET_Myc cells. **E** RNA Pol II traveling ratio. **F** Histograms and Box plots depicting H3K36me3 levels in WT and ∆SET cells. **G** Relative expression of *MYC* in *SETD1B* knockout MOLM-13 cells at day 4 post-Dox induction. **H** Histograms showing H3K4me3 and H3K36me3 levels in *SETD1B* knockout MOLM-13 cells. Gene loci annotated from the top 10% of H3K4me3 breadth (*n* = 915) in Fig. 4G were used. **I** Representative browser views of ChIP-seq showing H3K4me3 and H3K36me3 enrichment at the *MYC* locus in MOLM-13 cells. **P < 0.01, *P < 0.05, n.s. no significance.

H3K4me3 and MYC promote transcriptional elongation [28, 29, 33–35]. We performed ChIP-seq for RNA Pol II and Ser2 phosphorylation at the C-terminal domain of RNA Pol II (S2P) to monitor the effect on transcriptional elongation after reducing H3K4me3 breadth and MYC expression. The RNA Pol II signal was reduced at the gene body, but not the TSS, in ∆SET mutants (Fig. 6A, B). S2P was also significantly reduced in the gene body of the ∆SET mutant (Fig. 6A, C). MYC expression enhanced RNA Pol II recruitment to the TSS, but restoring RNA Pol II and S2P signals on the gene body was limited (Fig. 6A–C). The defective restoration of gene expression levels in MYC-expressing ∆SET cells was positively correlated with reduced S2P (Fig. 6D). The extent of MYC binding was not associated with the S2P levels in the gene body (Supplementary Fig. 3A). The comparable RNA Pol II traveling ratio between ∆SET and MYC-rescued ∆SET cells indicate the defective transcriptional elongation in MYC-rescued ∆SET cells (Fig. 6E). H3K36me3, a well-known transcriptional elongation marker, was also significantly decreased in ∆SET cells (Fig. 6F). To further investigate the association between H3K4me3 and H3K36me3 following the loss of SETD1B, we used doxycycline-inducible Cas9-expressing MOLM-13 cells. Consistent with the mouse leukemia model, the loss of SETD1B reduced the MYC expression and broad H3K4me3 peaks at 4 days post-Dox treatment (Fig. 6G–I). However, no significant reduction was observed SETD2 and H3K36me3 peaks (Fig. 6H–I, Supplementary Fig. 3B). These data suggest that the long-term reduction of H3K4me3 breadth may cause the severe transcription elongation defects accompanied by H3K36me3 reduction. We also evaluated the role of the SETD2/H3K36me3 axis using SETD2 knockout cells. Notably, SETD2 loss resulted in reduced cell numbers, H3K36me3 levels, and MYC expression in MOLM-13 cells (Supplementary Fig. 3C–E). Additionally, we observed elevated levels of H3K4me3 and H3K36me3 in primary FLT3-ITD mutated AML samples (Supplementary Fig. 3F). These results suggested that SETD1B is required for MYC expression and H3K4me3-mediated transcriptional elongation for cytokine-independent AML cell proliferation.

**Fig. 7:**
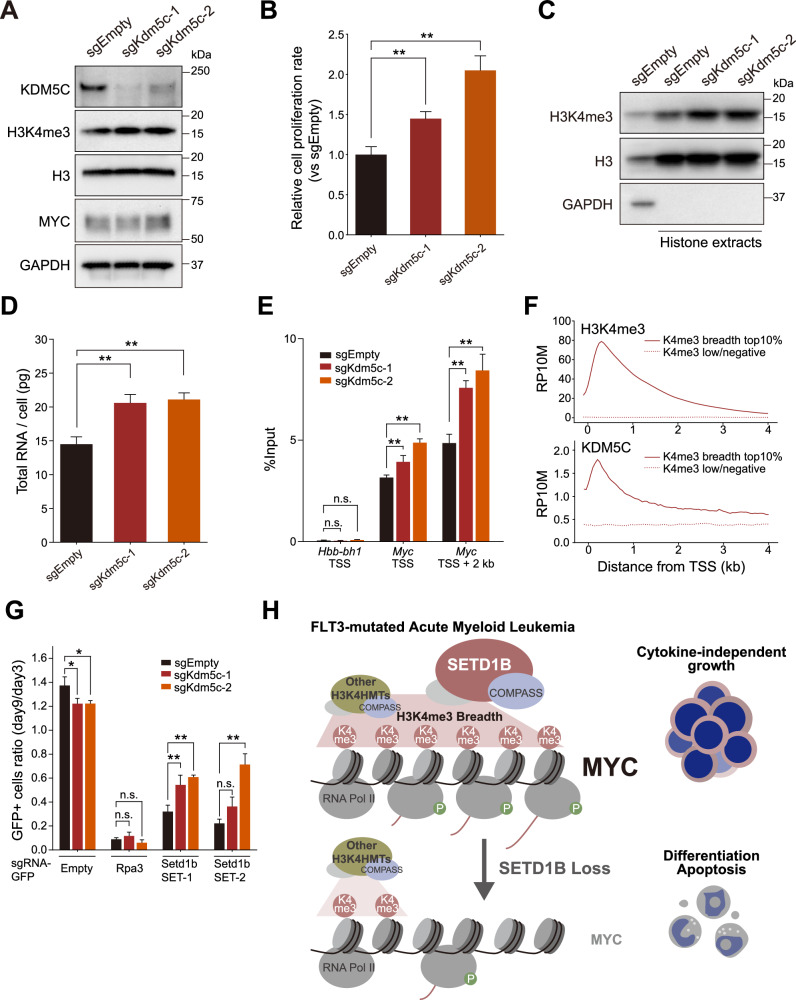
KDM5C demethylase regulates SETD1B-mediated H3K4me3 and MYC expression in AML cell proliferation. **A** Western blot analysis in *Kdm5c* sgRNA-expressing cells. **B** The relative cell proliferation rate of *Kdm5c* sgRNA-expressing AML cells. **C** Western blot analysis of histone extracts. **D** Total RNA from *Kdm5c* sgRNA-expressing AML cells. **E** ChIP-qPCR of H3K4me3 at *Hbb-bh1* and *Myc* loci. **F** Histograms of the average ChIP-seq signals of H3K4me3 and KDM5C at downstream from TSS in MOLM-13 cells. The analysis compares the breadth of top 10% genes (*n* = 915) and an equal number of randomly selected genes with low or undetectable H3K4me3 signals (*n* = 915). **G** CRISPR knockout of *Setd1b* in *Kdm5c* sgRNA-expressing cells and a competitive growth assay. The ratio of sgRNA-GFP-expressing cells was monitored on days three and nine post-infection. **H** A proposed model of cytokine-independent growth driven by SETD1B-mediated H3K4me3 expansion and MYC upregulation in FLT3-mutated AML.

We also disrupted the H3K4me3 demethylase *Kdm5c* to investigate the role of H3K4me3 in AML cell proliferation, which showed tumor suppressor activity in our CRISPR assays (Figs. 1A, 7A, B, Supplementary Figs. 1F, 4A). In wild-type leukemia cells, *Kdm5c* knockout increased H3K4me3 levels (Fig. 7C, Supplementary Fig. 4A). Although the MYC protein level was maintained, perhaps through tight regulation by the ubiquitin-proteasome pathway, we observed an increase in total RNA and enhancement of H3K4me3 at the *Myc* locus (Fig. 7D, E). We observed an overlap in the distribution of KDM5C with both SETD1B and broad H3K4me3 peaks in MOLM-13 cells (Fig. 7F and Supplementary Fig. 4B). Notably, *Kdm5c* knockout partially rescued the growth of ∆SET cells in a competitive growth assay (Fig. 7G). These data indicate that H3K4me3 is a key histone modification involved in transcriptional elongation and cell proliferation of MLL-r AML cells (Fig. 7H).

## Discussion

The redundant roles of SETD1A and SETD1B have been evaluated in embryonic stem cells [36, 37]. In this study, we provided evidence that the non-redundant SETD1B function regulates H3K4me3 breadth and supports the cytokine-independent growth in MLL-r AML cells. In MLL-r leukemia, SETD1A assumes a non-catalytic role in the transcriptional elongation of genes crucial for DNA repair and mitochondrial respiration [23, 24]. In contrast, our results suggested that SETD1B is a bona fide H3K4me3 methyltransferase in leukemia cells. Consistent with our data, SETD1B regulates broad H3K4me3 peaks in embryonic stem cells and neurons [37, 38]. A tumor suppressor role of KDM5C has also recently been reported in other leukemia subtypes [39]. Furthermore, H3K4me3 is integral in the RNA Pol II pause release and subsequent elongation [29]. Therefore, our study indicates that two Set1 homologs orchestrate the transcriptional elongation by their non-catalytic and catalytic roles in AML cells.

Similar to Set1 homologs, MYC plays a pivotal role in transcriptional pause release and elongation [13, 14, 28, 29, 33]. Consistent with the MYC function in global RNA synthesis and turnover [34, 35, 40], ∆SET cells exhibited a notable reduction in RNA quantity, which is restored by exogenous MYC expression. In contrast, H3K4me3 breadth in ∆SET cells was not recovered by MYC restoration. Consequently, while MYC ranks among the most critical downstream targets of SETD1B, preserving H3K4me3 breadth remains essential for augmenting global gene expression by MYC. Moreover, MYC expression was significantly reduced immediately following *SETD1B* knockout, while SETD2 and H3K36me3 levels remained unaltered. The breadth of H3K4me3 is crucial for transcriptional consistency, suggesting that MYC expression is highly dependent on both the quality and quantity of transcriptional elongation [13]. Therefore, a thorough understanding of the role of SETD1B-mediated H3K4me3 breadth is critical for developing markers and therapies for other leukemia subtypes and cancers dependent on MYC.

SETD1B is required in lymphoid-primed multipotential progenitors and lymphoid lineages in mice [41]. Genetic alterations in SETD1B have been linked to poor outcomes in patients with leukemia, including frameshift or nonsense mutations in chronic myeloid leukemia [42] and a missense mutation in the non-catalytic region (Ala889Asp) in chronic lymphoproliferative disorder of natural killer cells [42]. Recurrent mutations in SETD1B co-occur with STAT3 mutations in T-large granular lymphocyte leukemia [43]. Although the precise contribution of SETD1B mutations in leukemia prognosis remains unclear, these data and our study suggest the cell context-dependent role of SETD1B in hematopoiesis and leukemogenesis.

FLT3-ITD activates the T-cell factor (TCF)-dependent *Myc* promoter and enhances progenitor self-renewal in myeloid lineage cells [44]. Therefore, this oncogenic signaling may strongly depend on SETD1B. While the presence of FLT3-ITD mutation does not correlate with the expression of *SETD1B* transcription in AML patients, FLT3-ITD may promote SETD1B catalytic activity through cell signaling pathways, including the Ras signaling pathway. The role of SETD1B was also reported in RBM15-MKL1-fused AMKL cells [45]. RBM15-MKL1 stimulates the Notch signaling pathway that is required in T-cell leukemogenesis [46]. Since H3K4me3 breadth is linked to cell fate, the dependency on SETD1B could be modulated by specific oncogenes that induce an abnormal cell fate [13]. In addition, RBM15 interacts with SETD1B, and the fusion protein causes the cytokine-independent proliferation. RBM15 and its fusion protein regulate *Myc* expression in hematopoietic cells [47]. Thus, oncogenic driver mutations that induce cytokine-independent growth in leukemia would commonly depend on the SETD1B-MYC axis.

In conclusion, we identified the function of the catalytic domain of SETD1B in H3K4me3 breadth and cytokine-independent cell proliferation in MLL-r leukemia. Our study also highlights the crucial role of SETD1B-dependent H3K4me3 breadth in the MYC pathway in leukemia. Despite the extensive experiments we conducted, we acknowledge that the efficacy of SETD1B suppression by inhibitor and its potential side effects in vivo remain unknown. The development of a SETD1B-specific inhibitor or an expanded application of HMT inhibitors, such as Chaetocin, which also target SETD1B, will be a valuable tool against MYC-dependent leukemia [48].

## Supplementary information

Supplemental Figures

Suppmental Table

## Data Availability

The accession numbers for the RNA-seq, ChIP-seq, and CUT&Tag data reported in this paper are NCBI GEO GSE248326. Previously deposited data (GSM5737309 and GSM5737312 in GSE189894) were used in this study.
