# Supplementary material for: Regulation of H3K4me3 breadth and MYC expression by the SETD1B catalytic domain in MLL-rearranged leukemia
Source: Leukemia. 2025 May 8;39(7):1627–39. doi: 10.1038/s41375-025-02638-y (PMC12208907; doi:10.1038/s41375-025-02638-y)
Supplement: Supplementary file 1 — Supplemental Figures [file 41375_2025_2638_MOESM1_ESM.docx]

**Supplemental Data**

**Regulation of H3K4me3 breadth and MYC expression by the SETD1B catalytic domain in MLL-rearranged leukemia**

Correspondence to: hoshiit@chiba-u.jp

**This PDF file includes:**

Supplemental Figure 1 to 4

Supplemental Table 1

List of DNA, RNA and antibodies used in this study.

**Supplemental Figures**

**
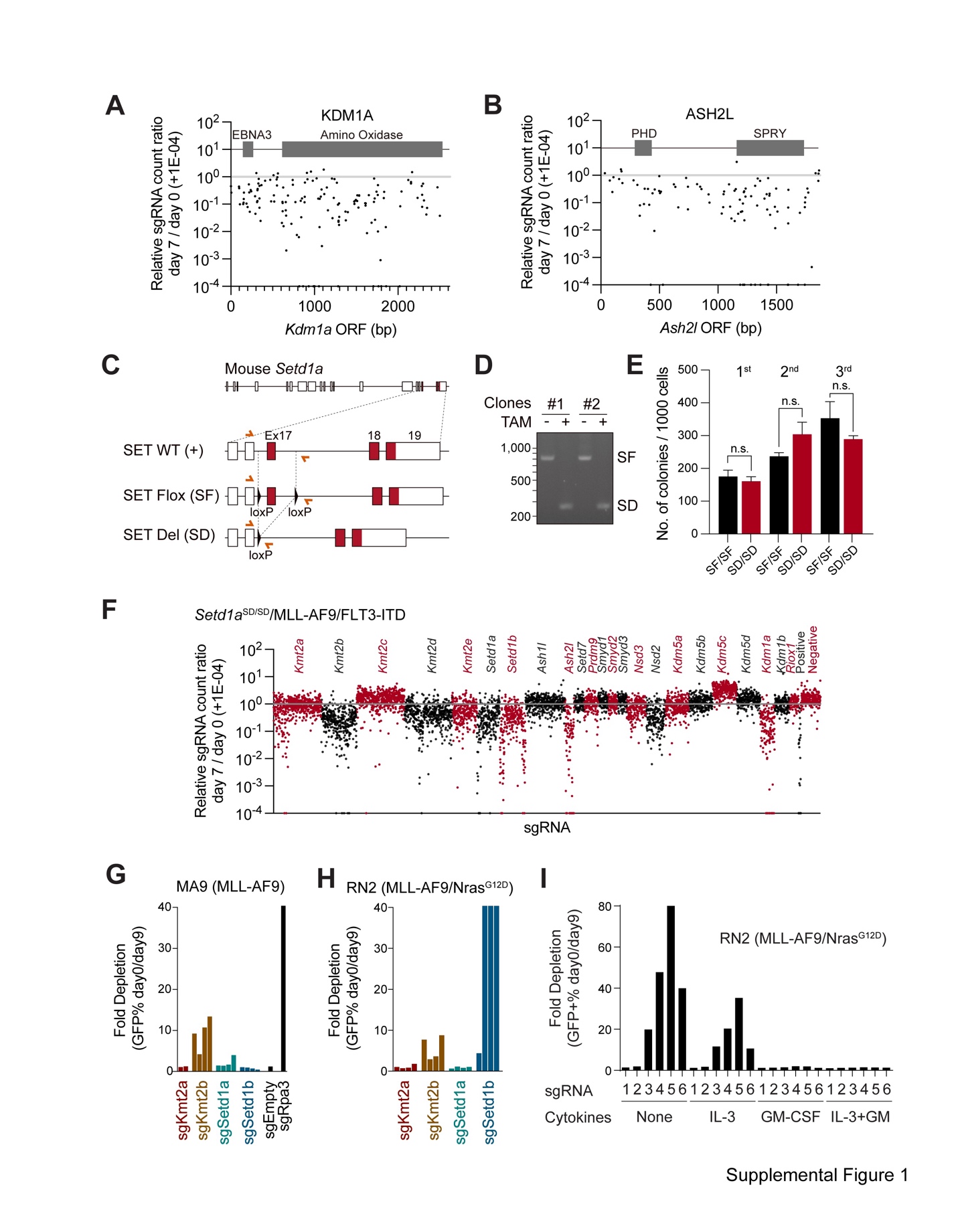
**

**Supplemental Figure 1. Establishing *Setd1a*^SF/SF^, MLL-AF9, FLT3-ITD, and CreER^T2^ AML cells.**

(A–B) Relationship between known domains and sgRNAs against (A) *Kdm1a* and (B) *Ash2l*. Dots represent the relative sgRNA count ratios from the CRISPR tiling screen in MLL-AF9/FLT3-ITD/Cas9 AML cells. (C) Schematic representation of the conditional knockout allele of *Setd1a* exon 17 in mice. Regions encoding the catalytic domain of SETD1A are shown in red. (D) PCR genotyping of *Setd1a*^SF/SF^, MLL-AF9, FLT3-ITD, and CreER^T2^ AML cells. (E) Serial replating colony formation assay. *Setd1a*^SF/SF^ and *Setd1a*^SD/SD^ AML cells in Figure S1D were cultured and replated thrice. n.s. = no significance. (F) Results of the pool CRISPR tiling screen against H3K4HMT genes in *Setd1a* exon 17 deleted cells with Cas9. (G–H) Individual sgRNAs against the Kmt2a, Kmt2b, Setd1a, and Setd1b SET domains were transduced into (G) MA9 (MLL-AF9 with IL-3) and (H) RN2 (MLL-AF9/Nras^G12D^ without IL-3) cells with Cas9, and competitive growth assays were performed. Rpa3 sgRNA was used as a positive control. (I) Negative selection experiments evaluating *Setd1b* SET sgRNA in RN2 MLL-AF9/Nras^G12D^ cells were performed with or without cytokines.

**
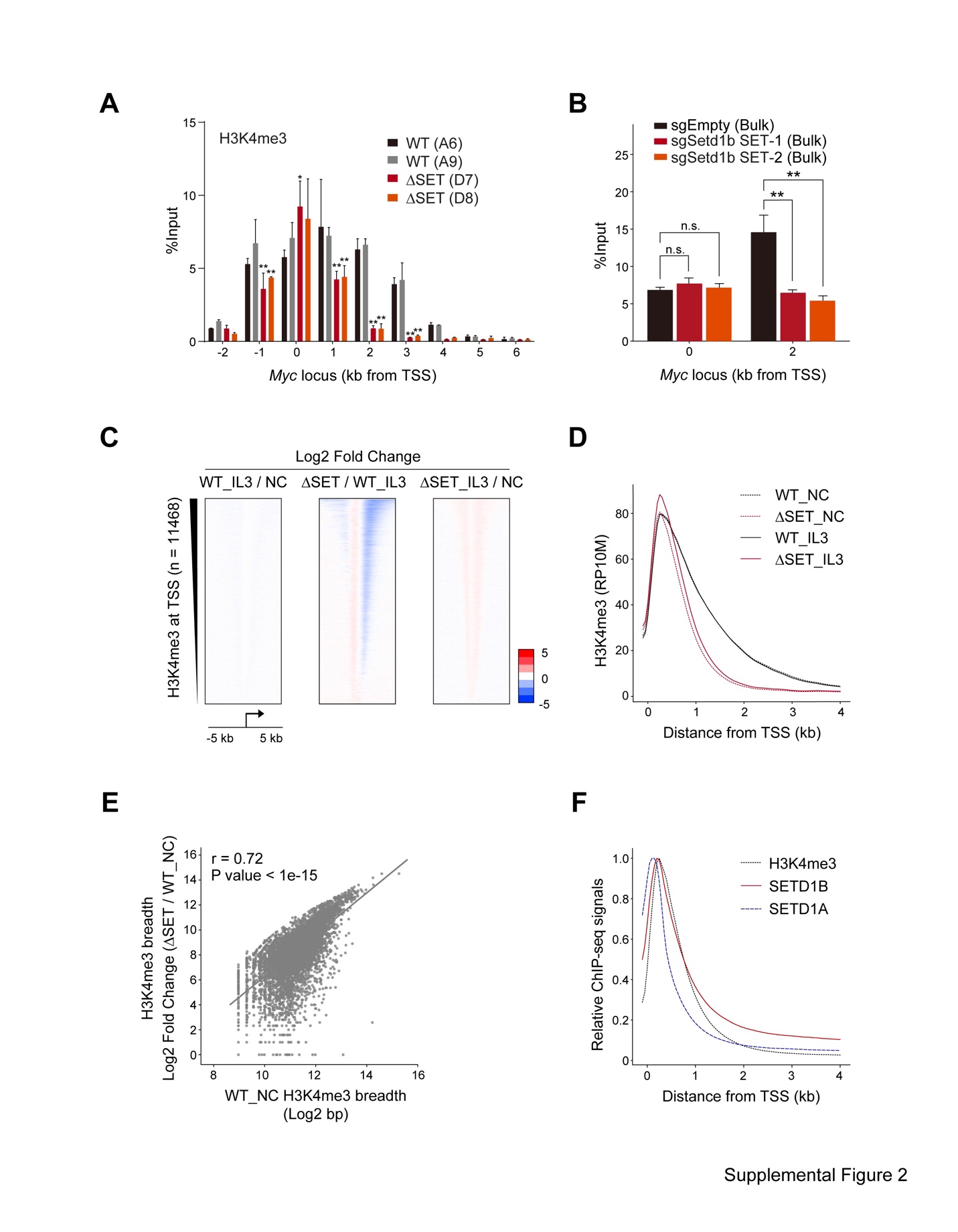
**

**Supplemental Figure 2. SETD1B is required for broad H3K4me3 at the gene body of the *Myc* locus.**

(A) The ChIP-qPCR results for H3K4me3 at *Myc* locus are shown. Two independent clones from wild-type (WT) and ∆SET AML cells were used. (B) ChIP-qPCR for H3K4me3 at the *Myc* locus in sgRNA-expressing MLL-AF9/FLT3-ITD AML cells is shown at day 10 post-sgRNA infection. (C) Heatmaps of log2 fold changes of H3K4me3 signals among indicated samples are shown. (D) The histograms of H3K4me3 signals at downstream from TSS are shown (n = 11468). (E) Spearman’s rank correlation between the original H3K4me3 breadth in WT cells and the log2 fold change of decreased H3K4me3 breadth in ∆SET cells are shown. (F) The histograms of relative ChIP-seq signals of H3K4me3, SETD1B (HA-tag) and SETD1A (HA-tag) are overlayed. Signals were normalized by the maximum intensity of each factor. **P < 0.01, n.s. = no significance. TSS, transcription start site


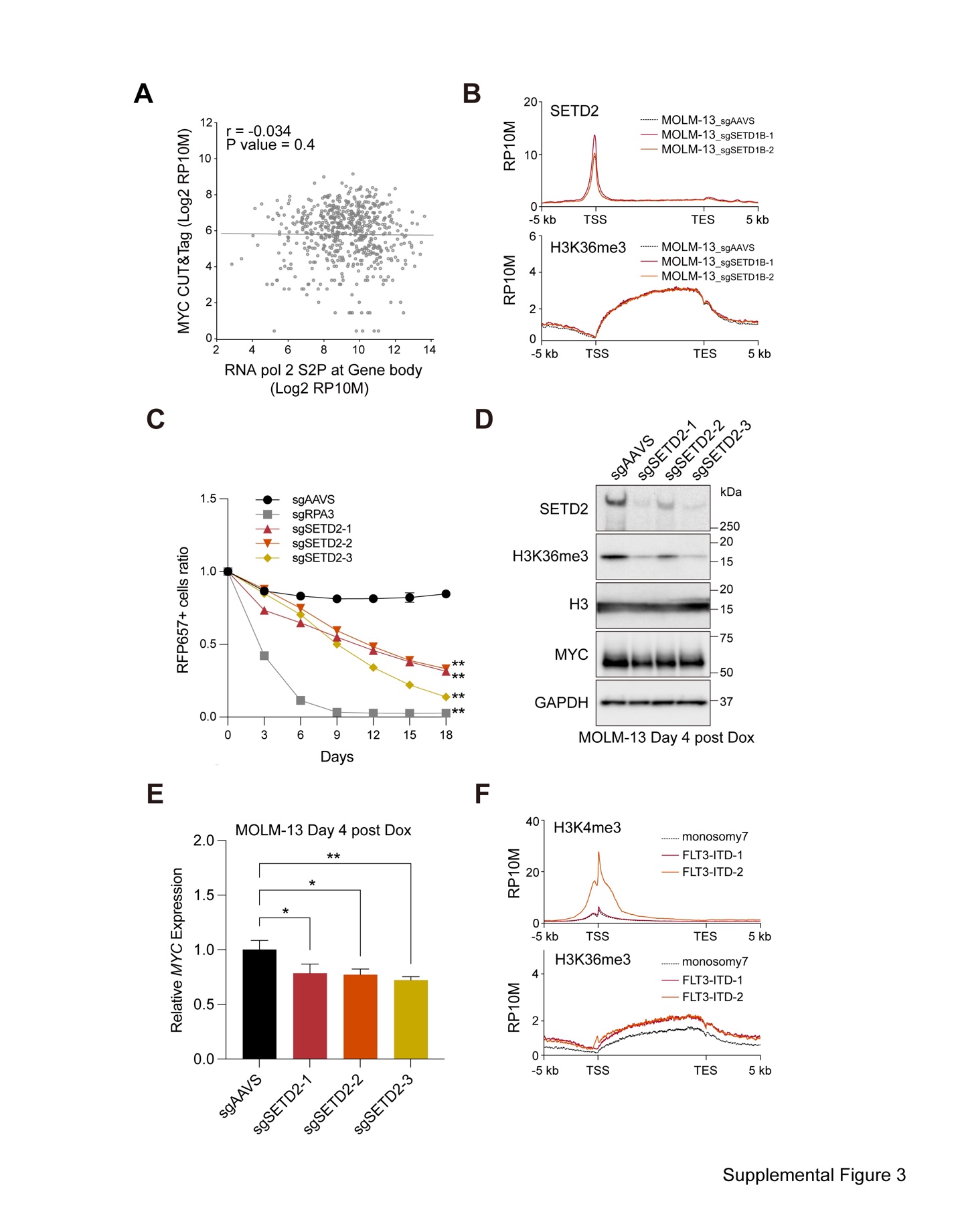


**Supplemental Figure 3. SETD2/H3K36me3-mediated MYC expression in AML cells.**

(A) No positive correlation was observed between the RNA Pol II S2P level and MYC binding. (B) Histograms of SETD2 and H3K36me3 levels in SETD1B knockout MOLM-13 cells. (C) The competitive growth assay of *SETD2* sgRNA-expressing MOLM-13 cells. *RPA3* sgRNA was used as positive control. (D) Western blot analysis in *SETD2* sgRNA-expressing cells day 4 post-Dox induction. (E) The relative expression of MYC in *SETD2* sgRNA-expressing cells at day 4 post-Dox induction. (F) Histograms of H3K4me3 and H3K36me3 in primary AML samples. One sample from monosomy 7 AML and two samples from FLT3-ITD AML were compared. *P < 0.05, **P < 0.01.

**
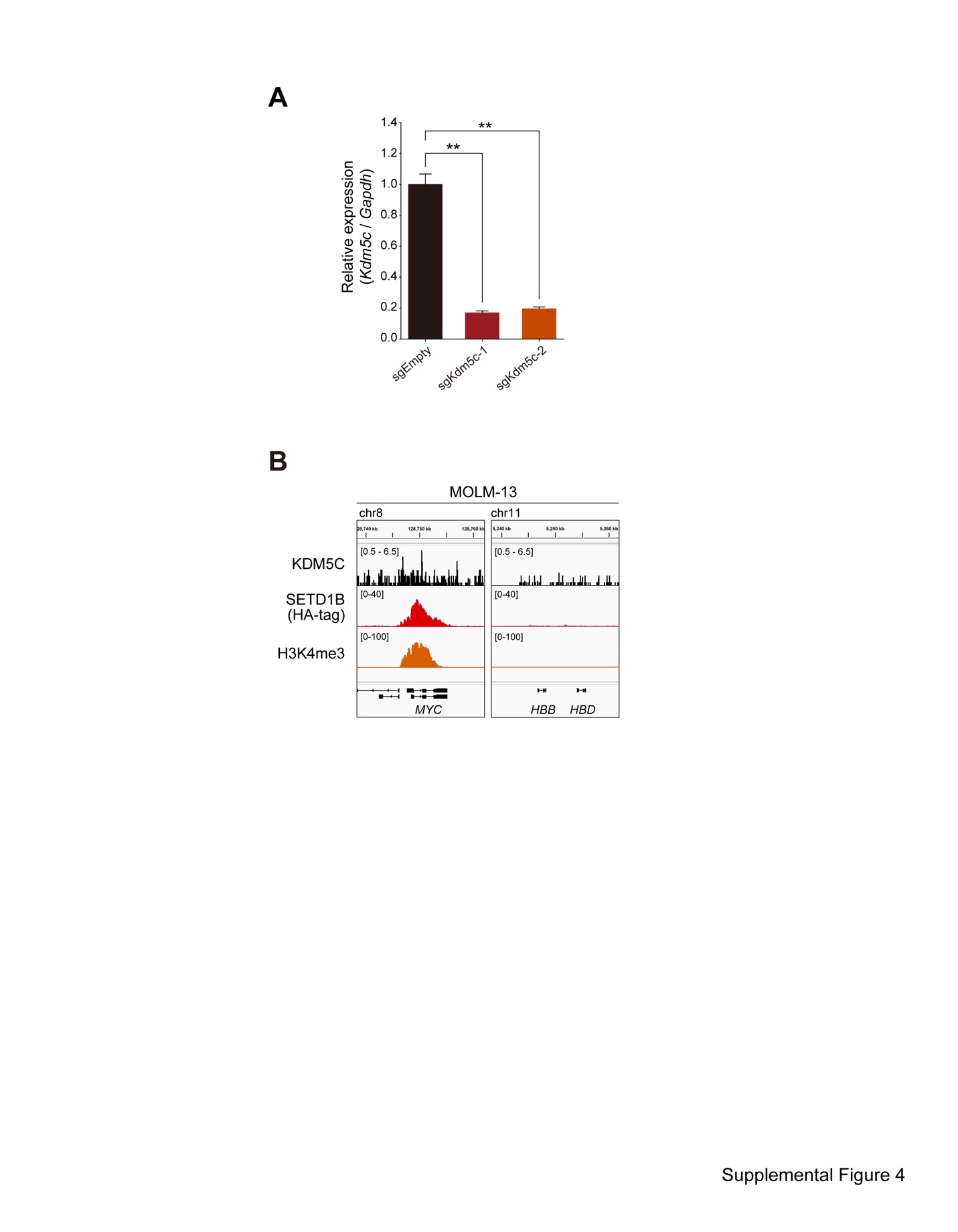
**

**Supplemental Figure 4. KDM5C suppresses H3K4me3 and AML cell proliferation.**

(A) The relative expression of *Kdm5c* in *Kdm5c* sgRNA-expressing AML cells. (B) Representative browser views of ChIP-seq for KDM5C, SETD1B (HA-tag) and H3K4me3 at *MYC* and *HBB*/*HBD* loci in MOLM-13 cells. *HBB*/*HBD* locus was shown as negative control. **P < 0.01.
